# Thermal Stability and Heat Transfer of Polyurethanes for Joints Applications of Wooden Structures

**DOI:** 10.3390/molecules29143337

**Published:** 2024-07-16

**Authors:** Paweł Rutkowski, Konrad Kwiecień, Anna Berezicka, Justyna Sułowska, Arkadiusz Kwiecień, Klaudia Śliwa-Wieczorek, Boris Azinovic, Matthew Schwarzkopf, Andreja Pondelak, Jaka Gašper Pečnik, Magdalena Szumera

**Affiliations:** 1 Department of Ceramics and Refractories, Faculty of Materials Science and Ceramics, AGH University of Krakow, al. A. Mickiewicza 30, 30-059 Krakow, Poland; berezicka@agh.edu.pl (A.B.); sulowska@agh.edu.pl (J.S.); 2 Department of Biomaterials, Faculty of Materials Science and Ceramics, AGH University of Krakow, al. A. Mickiewicza 30, 30-059 Krakow, Poland; kkwiecien@agh.edu.pl; 3 Chair of Structural Mechanics and Material Mechanics, Faculty of Civil Engineering, Cracow University of Technology, Warszawska 24 Street, 31-155 Krakow, Poland; arkadiusz.kwiecien@pk.edu.pl; 4 Chair of Bridge, Metal and Wooden Structures, Faculty of Civil Engineering, Cracow University of Technology, Warszawska 24 Street, 31-155 Krakow, Poland; klaudia.sliwa-wieczorek@pk.edu.pl; 5 Slovenian National Building and Civil Engineering Institute, Dimičeva 12, 1000 Ljubljana, Slovenia; boris.azinovic@zag.si (B.A.); andreja.pondelak@zag.si (A.P.); 6 InnoRenew CoE, Livade 6a, 6310 Izola, Slovenia; matthew.schwarzkopf@innorenew.eu (M.S.); jaka.pecnik@innorenew.eu (J.G.P.)

**Keywords:** polyurethanes, FPU, RPU, adhesives, thermal conductivity, thermal expansion, thermal diffusivity, thermal stability

## Abstract

Wood characterized by desired mechanical properties and wood joining material is essential for creating wooden structures. The polymer adhesives are suitable for such applications due to the possibility of energy dissipation from stresses generated by wooden structures and the elimination of thermal bridging, which are common problems in metal joining materials. This research focuses on the thermophysical properties of the laboratory-prepared flexible and rigid polyurethanes to select an appropriate polymer adhesive. Our results showed that the highest thermal stability was in the case of the new PSTF-S adhesive, which reached 230 °C, but the lowest mass loss in the air environment was around 54% for the PS material. The mean thermal expansion coefficient for F&R PU adhesives was 124–164∙10^−6^ K^−1^. The thermal diffusivity of examined adhesives varied between 0.100 and 0.180 mm^2^s^−1^. The thermal conductivity, depending on the type of polyurethane, was in the 0.13–0.29 W∙m^−1^∙K^−1^ range. The relative decrease in thermal diffusivity after heating the adhesives to 150 °C was from 2% for materials with the lowest diffusivity to 23% for the PU with the highest value of heat transfer. It was found that such data can be used to simulate wooden construction joints in future research.

## 1. Introduction

In currently used timber construction practices prevalent in the Northern Hemisphere, the utilization of the softwood species predominates. This is evidenced by the fact that glued laminated timber is primarily composed of softwood laminations. Nevertheless, there has been a growing recognition of the potential applicability of hardwood species, namely ash, oak, or beech, traditionally employed in non-structural applications as valuable timber resources for construction purposes [1]. The significantly higher average density of these tree species, as compared to their softwood counterparts, translates to significantly improved mechanical properties. Another reason for this increasing interest in hardwood is climate change coming from human interference in natural ecosystems. It is expected that, due to the warmer winters, tree species distributions may change in the areas of western and central Europe, and consequently, indigenous conifers may be replaced by the more competitive deciduous trees, beech being one of them [2]. Hence, because the demand for timber has been steadily increasing in recent years, which subsequently leads to competition among various wood-consuming sectors [3], efforts are made to seek solutions that promote sustainable utilization of these resources.

The proactive approach toward addressing the requirements for environmental sustainability has resulted in significant advancements in adhesive technology, enabling the utilization of smaller-dimensioned timber as a substitute for solid wood in various structural and non-structural applications. Worth emphasizing remains the fact that adhesively bonded materials display improved performance compared to not only solid wood but also mechanically connected timber structural elements. This is because the conventional joining method is bound to the thermal bridge generation due to the high thermal conductivity of metal fixing parts. This contributes to the wood fibers’ disruption, entails significant costs, and frequently allows for water infiltration into the wooden structure [4]. Simultaneously, adhesively bonded joints are recognized for their enhanced homogeneity and predictable properties, with their performance being less susceptible to the variations in the characteristics and flaws of each piece of wood [5].

Nevertheless, the pursuit of environmental neutrality has led to an increasing preference for adhesives characterized by low or no formaldehyde emission, resulting in a systematic displacement of conventional adhesive systems by polyurethanes [6]. These peculiar materials are renowned for their strong adhesion to a wide range of substrates, primarily attributed to the presence of polar HNCOO groups with high cohesive energy. They also exhibit remarkable elasticity, especially when combined with various additives, and demonstrate significant chemical durability, including moisture resistance. It is also of considerable significance that polyurethane adhesives possess the ability to withstand prolonged vibrations and significant impact stresses [7]. On the other hand, the key characteristic of polyurethanes is the possibility to customize their properties to a wide extent by making alterations to the molecular chain structure of both the soft (consisting of long polyols) and the hard (formed by short diols and isocyanates) segments, building the PUR’s framework [8]. This customization is of paramount importance in the context of glued wood products, as the adhesive material must closely match the properties of the wood component, particularly under the conditions of the product application.

Therefore, given the fact that a vast majority of glued wood products are intended for outdoor use or building constructions, and in the temperate climate of Central Europe, large daily temperature fluctuations are observed; it is the thermal properties of the bond line that should be carefully considered to not affect the integrity of a wooden structure.

One of the requirements imposed on macromolecular compounds such as polyurethanes is to exhibit thermal stability not only during processing but also throughout the whole application. Consequently, to ensure the durability of an adhesive under conditions of exposure to seasonally variable temperatures, the emphasis should be placed on exploiting polymers that possess distinct features, signaling their enhanced thermal stability, i.e., elevated melting, softening, and thermal decomposition temperatures, reduced mass loss, negligible alterations in their mechanical, physical, and chemical properties when subjected to elevated temperatures, as well as higher heat deflection temperature under load [9]. It is worth noting that the evaluation of these indicators of improved thermal stability can be accomplished through standard thermal analysis techniques, including Thermogravimetric Analysis (TGA) and Differential Scanning Calorimetry (DSC).

The techniques mentioned above are commonly used to test the thermal properties of various adhesives dedicated to wood. Hong et al. [10] used TGA and DSC methods to analyze a modified polyvinyl acetate adhesive. Thanks to their modifications with N,N-dimethylethylenediamine and sulfanilamide, they were able to not only improve the adhesive properties but also change the thermal decomposition manner that went in one stage instead of two at temperatures exceeding 300 °C for all the tested heating rates. However, a detailed thermal analysis is required. Balkova et al. [11] compared a few adhesives regarding their thermal properties. The chosen epoxy resin decomposed thermally above 340 °C. However, the four-point bending test of lap-jointed spruce specimens showed that they lose mechanical strength significantly after heating to 140 °C even for 20 min.

On the contrary, the tested PUR adhesive showed the highest strength after exposure to 140 °C. However, when kept at 170 °C for over 80 min, the strength rapidly decreased, although it did not show decomposition signs up to 200 °C. Our preliminary studies of a chosen PUR adhesive [12] also showed the thermal stability of the polymer above 200 °C and no significant loss of strength after exposure to an alkali aqueous environment for 1000 h. On the other hand, the influence of higher temperatures on the different features of the material was not tested. Minding the increased temperatures in various regions of the world, strict regulations for the construction materials against fire, and the ecological aspect of reducing heat losses, thermal analysis of the adhesives should be provided for all the novel materials for this purpose.

According to Prociak et al. [13], the thermal characteristics of high-molecular-weight polymers may be adequately assessed only by determining both thermal conductivity and thermal diffusivity. Nevertheless, considering the significant influence of various factors, including chemical composition, structural configuration, bond strength, molecular weight of the side groups, and processing conditions, it is not surprising that there is a substantial variability in the reported experimental data for the thermal conductivity and diffusivity of the crystalline and semi-crystalline polymers [8]. Also noteworthy is the fact that the vast majority of available data addresses the mentioned parameters in the context of polyurethane foam insulations [13,14]. Despite that, there are indications that the thermal conductivity generally shows an upward trend as the content of chain extender added during the synthesis of the polyurethanes increases, thereby suggesting the possibility of manipulating this parameter to achieve the desired properties in the final product [8].

In every bonded joint, thermal stresses arise from the disparate thermal expansion characteristics between the adhesive and the adherents, as well as from the adhesive shrinkage during the curing process. Remarkably, as indicated by [15], the stresses stemming from the phenomenon of adhesive shrinkage exert a considerably lesser impact on the joint strength compared to those induced by the thermal mismatch of the adherents. It is often acceptable to disregard the thermal stresses arising from the adherend adhesive thermal mismatch when bonding similar adherents. Worth mentioning here are the results of the study of [16], who showed that a relatively soft polyurethane adhesive can compensate for the gap changes generated by the materials with different thermal expansion coefficients. Nonetheless, the utilization of adhesively bonded materials under conditions of high seasonal temperature variability makes it crucial to ensure that the adhesive’s coefficient of thermal expansion is compatible with that of the wooden component [17].

Finally, when selecting a material for adhesive bonding, careful consideration must be given to the influence of cyclic thermal loads, as they can induce debonding phenomena and ultimately lead to joint failure. The recently developed approach promotes bonding solutions that facilitate suitable material performance in the presence of cyclic or dynamic (e.g., seismic) loads. Consequently, the search for solutions incorporating low-stiffness polyurethanes, which would diminish stress concentrations and enhance both ductility and energy dissipation capacity, is still ongoing [17].

The significance of adhesives’ mechanical properties is widely acknowledged, given their role in transferring mechanical stresses between adherents. However, in the context of adhesive application within the variable climate of Central Europe, its thermal properties assume heightened importance, as the response of the bond line to the temperature fluctuations can exert an influence on the structural integrity of the wooden components. In light of the above, the present study aimed to determine the thermophysical properties of various polyurethane adhesives to select the most suitable one for creating joints with wooden elements characterized by the highest thermal stability. To address this objective, both flexible and rigid polyurethane adhesives were prepared and subjected to comprehensive thermal analysis, including thermal expansion and heat transfer evaluations. It is worth emphasizing that these specific polyurethane formulations have not yet been described in the literature, thus this work expands the knowledge base regarding adhesive bonding for the wooden structures. Furthermore, the analysis of the thermal stability, heat flow, thermal expansion, thermal diffusivity, and thermal conductivity would provide fundamental data for future research, simulations, and the selection of appropriate adhesives for joining wooden structures.

## 2. Materials and Methods

The polyurethanes were prepared in a way that allowed us to obtain materials dissipating vibration energy in wooden structures, with good thermal stability, and the ability to absorb the sound. They showed both flexible and rigid behavior. Two kinds of adhesives (FlexAndRobust Systems Sp. z o.o., Krakow, Poland F&R) were used:

- F&R PS, which is a solvent-free, elastic, two-component, polyurethane-based adhesive. It is used to join structural parts together and to prepare protective coatings.
- F&R PM, which is solvent-free, flexible, and of high-quality recovery. It is a two-component polyurethane-based adhesive.

**Figure 1:**
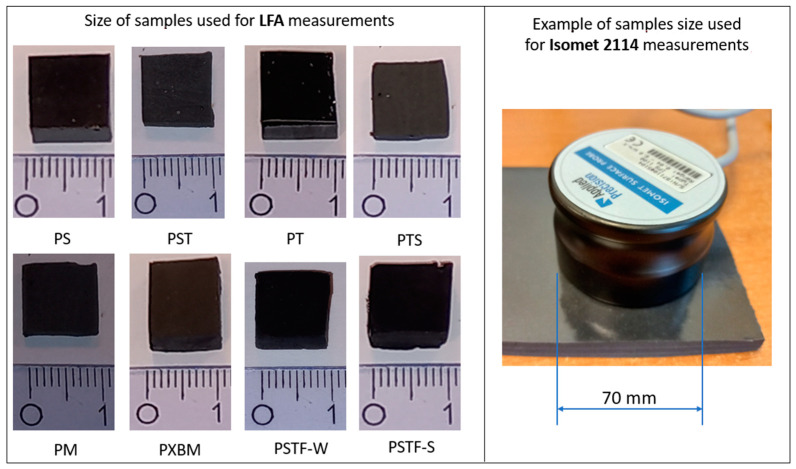
Comparison of flexible and rigid PU used for investigations by LFA and Isomet 2114 analysis.

After mixing the components, 280 mL of adhesives-based materials were poured into the molds to obtain samples in the size of 400 mm × 70 mm × 10 mm. The material description (abbreviation, components ratio, features, and density, given by the manufacturer) is collected in Table 1. The collected polyurethane adhesives used in constructing patented technology of Polymer Flexible Joints are covered by patent [18]. The various A:B given in the table is a ratio of the major resin component (such as F&R PS) to the hardener, which allowed us to modify the adhesives’ properties. The visual comparison of the prepared materials for thermal properties investigations by laser flash analysis (LFA) method and Isomet 2114 is presented in Figure 1.

The so prepared materials were taken into thermal analysis by differential scanning calorimetry (DSC) and thermal stability measurements using thermogravimetry (TG). DSC and TG measurements were performed using the DSC/TG carrier on STA 449 Jupiter F3 of Netzsch (Selb, Germany). The analysis was carried out in 40 mL·min^−1^ synthetic air flow in aluminum crucibles with a 5 °C∙min^−1^ heating rate. The final temperature was 600 °C. The recorded data was analyzed using mass change (TG), the first derivative of the mass change (DTG), and the heat flow. The major thermal effects were described by the onset and end set temperatures and the reaction heat as well.

For wooden constructions, thermal expansion of polymers is also very important. This is why all of the FPU- and RPU-based materials were subjected to dilatometric measurements, which were performed on up to 5 samples to obtain an average value. The examinations were made by the DIL 402C dilatometer of Netzsch (Selb, Germany). The measurement was provided on 6 mm diameter samples with the length close to 25 mm. The measurement was made in static conditions with an air heating rate of 1 Cmin^−1^ up to 80 °C.

Thermal diffusivity, volume heat capacity, and thermal conductivity were determined by the Isomet 2114 apparatus [19] on the large samples in a size around 400 mm × 70 mm and thickness from 5–15 mm. The samples were measured by type IPS 1100 surface probe and in the 0.04–0.3 W∙m^−1^∙K^−1^ measurement range. The accuracy of thermal conductivity measurement was 5%, and measurement reproducibility was 3%. Isomet 2114 allows the investigation of thermomechanical properties in dynamic conditions. During the measurement, the heating element based on the resistor generates heat flow in the sample. This results in the temperature response of the sample. The temperature monitoring of the sample in the function of time allows the calculation of thermal conductivity, thermal diffusivity, and volume heat capacity [19]. The validity of this method was also emphasized in the literature when comparing stationary and non-stationary measurement methods [20]. The device records the heat per unit volume and temperature changes and calculates the volumetric heat capacity C_p_. Having material density, it is possible to calculate specific heat capacity (c = C_p_/g) [21]. The following equation can calculate thermal diffusivity:

(1) $$a=\frac{\lambda }{volc_{p}}$$

where:

λ—thermal conductivity (W∙m^−1^∙K^−1^)
a—thermal diffusivity (m^2^∙s^−1^)
vol c_p_—volume heat capacity (J∙m^−3^∙K^−1^)

The homogeneity of the thermal diffusivity of such a large sample was checked on a statistically selected area of the material, from which samples 10 mm × 10 mm × 2.5 – 4 mm were subjected to laser flash analysis (LFA) examinations made by the LFA 427 of Netzsch (Selb, Germany,) company. In the laser flash analysis LFA method, direct thermal diffusivity is measured and calculated using the Cape–Lehman [22] and Cowan [23] models. The models were applied statistically, matched, and verified. Measurement is made by heating the material’s front surface with a short pulse of the laser and then recording temperature by an IR detector on the rear face of the sample. The measurement uncertainty was below 0.7%. The measurements were made 3 times per temperature. The material was investigated in static air at 50 °C, 100 °C and 150 °C.

The thermal measurements (DSC-TG-DTG, DIL, and LFA) were made at the Faculty Laboratory of Thermophysical Measurements at the Faculty of Material Science and Ceramics AGH University of Science and Technology (AGH University of Krakow).

## 3. Results and Discussion

### 3.1. Thermal Stability of Polyurethane Adhesives

Polyurethane adhesives are known for their excellent thermal stability compared to many other types. Their performance under various temperature conditions is due to their chemical structure and composition. Temperature resistance is undoubtedly a critical issue. Polyurethane adhesives generally have good resistance to a wide range of temperatures, both high and low. Depending on the specific formulation and additives, they can withstand temperatures from −40 °C to 150 °C or higher. It is also worth remembering the chemical structure of polyurethane adhesives contributes to their stability. They consist of urethane links, which are relatively stable and maintain their integrity even under elevated temperatures. Many polyurethane adhesives are formulated to undergo crosslinking reactions when cured. This crosslinking enhances thermal stability by creating a three-dimensional network that improves resistance to heat and chemicals.

**Figure 2:**
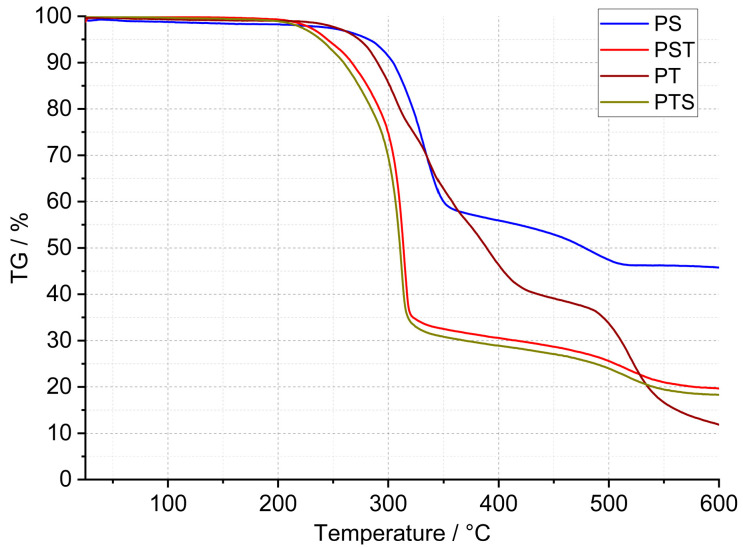
TG curves of PS, PST, PT, and PTS polyurethanes.

**Figure 3:**
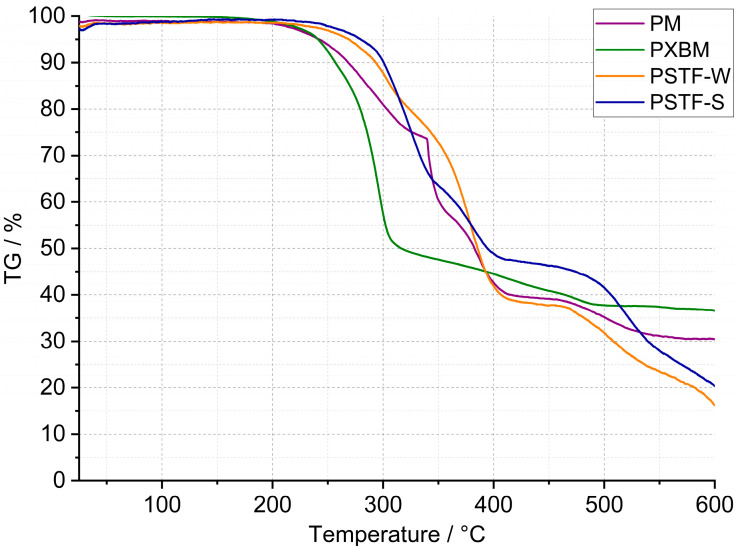
TG curves of PM, PXBM, PSTF-W, and PSTF-S polyurethanes.

**Figure 4:**
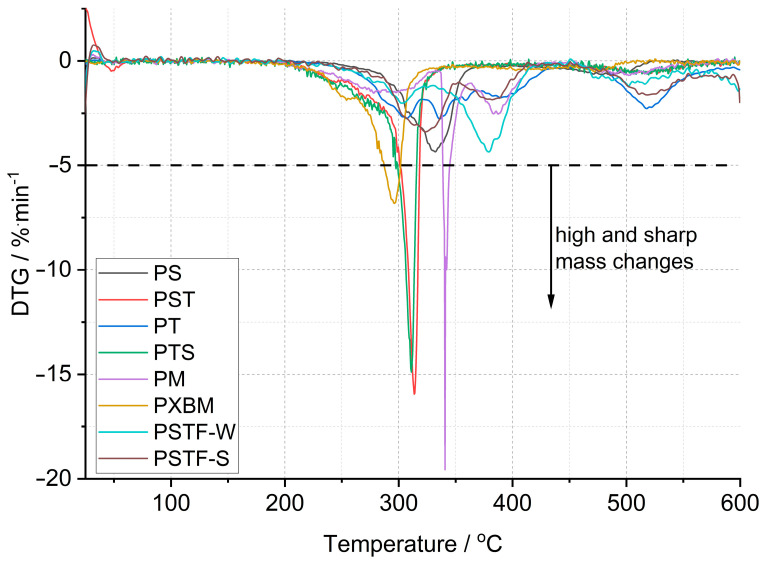
Comparison of the first derived mass changes in polyurethanes.

In the case of the PU presented in this work, curried-out thermal analysis up to 600 °C was made on several samples of each polyurethane, and the representative results are shown in Figure 2, Figure 3 and Figure 4. Detailed data on the heat flow and thermogravimetric analysis are collected in Table 2.

The thermogravimetric curves showed in Figure 2 and Figure 3 that the PT, PSTF-W, and PSTF-S samples decomposed losing 80–88% of their weight. The lowest mass loss is in the case of the PS sample, and it reaches 54%. This data is very important concerning fire events and related gases released. The PST and PTS samples behave almost identically. The data recorded on the thermogravimetric curves showed also that the polymer degradation in elevated temperatures consists of 2–4 steps. That mass loss quantity is an important thing in the case of materials applied in wooden constructions, but the mass change rate is much more significant due to the heat exothermic effect and environment ventilation rate of the released gases.

The first derivative (Figure 4) of mass changes shows that the PM, PST, and PTS are characterized by the strongest and violent effect, which cannot be desired in case of a fire event. The mass change rate reaches even 10–20%∙min^−1^, which can be too fast for gas ventilation in the fire area. In the case of the other polyurethane adhesives, the mass loss rate is at least 3 times lower. The PS sample showed, in connection with the lowest material mass loss, a low degradation rate. This material is also characterized by good thermal stability that reaches 200 °C, which is presented in Table 2. The lowest thermal stability was equal to 160 °C and was recorded for the PXBM material, which showed visually to be very soft/elastic and degraded at higher temperatures. The data collected in Table 2 indicate that the highest thermal stability, temperature without any mass changes, was recorded for the PSTF-S adhesive and it was even up to 230 °C. For most of the investigated polyurethanes, the thermal stability was in the 190–210 °C range. This table also presents the data of mass changes above the thermal stability temperature. The data in the table shows that the PS, PST, and PTS polymers reveal only two steps of mass changes and among them, the PS sample has the lowest values even in the first step. What is also significant is that the new PSTF-W and PSTF-S adhesives have high thermal stability, low mass change rate, and three decomposition steps not exceeding 40%, so the highest one was recorded in the case of the PS adhesive. The highest mass change was noticed for the PT material.

Concerning PU thermal analysis, there is a large amount of literature data and various PU-based materials, implicating change in onset degradation temperature. The result depends on PU modification and analysis of atmosphere/conditions. In the case of PUR containing polyglycerols and polyol, the mass changes start at a low temperature, much below 200 °C, linked with the curing process [24]. They confirmed that the significant degradation temperature starts at 200 °C, which is typical in literature. Usually, in literature, there are three stages of this process. This publication also says that the first degradation step is usually associated with the decomposition of hard segments and urethane linkages. The last two steps of this process are connected to soft segments of PU. Another paper, where PU is modified by, for example, siloxane, showed that initial decomposition at about 270 °C was explained by the decomposition of small molecules and carbamate groups and at higher temperatures by oxidation of methyl and phenyl groups. This last step caused CO/CO_2_ gas release and mass drops [25]. Also, in the case of bio-additives to PU, the thermal decomposition becomes above 200 °C with two major mass loss steps. The first step is connected with CO_2_ release coming from [25]. All our materials are composed of rigid and flexible components, which have various tasks in the wooden joint. Following the literature, rigid PU decomposes, breaking up urethane into isocyanate segments, and in the case of polyol segments, the degradation starts from about 200 °C. In higher temperatures, polyols give a product of aliphatic ether alcohol. In the temperature range of 350–500 °C, the following reaction products are recorded in literature: primary and secondary amines, vinyl ethers, and CO_2_ [26]. Flexible PUs are mostly used for foams but not adhesives, as in our case, and applications and thermal analysis are usually focused on the decomposition of such materials [27]. This paper indicated two processes: pyrolysis in nitrogen flow and oxidation in airflow. The first one, divided into two stages, concerns regenerated polyols and gaseous isocyanates products, and products of polyols into char + OH Species, H_2_CO, CH_4,_ and H_2_O. The second one has three stages concerning our case of analysis, as follows [27]:

- PU foam degradation to regenerated polyol + OH Species, CO_2_, and H_2_O;
- Regenerated polyol decomposes to char + OH species, H_2_CO, CH_4_, CO, CO_2_, and H_2_O;
- Char in reaction with oxygen gives OH species, H_2_CO, CH_4_, CO, CO_2_, and H_2_O products.

These data (Table 2) are also important to compare the thermal properties and auto-ignition of beech wood. The latest and most recent research on spontaneous ignition is from 2023 [28,29]. The authors showed a direct dependence between heating rate, the rise of the material temperature, the volume of beech wood, and its auto-ignition. An increase in the temperature and the material volume will decrease the onset of the ignition process. The initiation of this process for beech falls at 240 °C [28]. Ignition is aided by the decomposition of organics, such as hemicelluloses and cellulose, and a reaction on the surface of the wood. Therefore, it is very important to maintain the highest thermal stability of the polyurethane adhesive and the smallest exothermic and mass effects associated with the decomposition of PU (Figure 2 and Figure 4). The reactions associated with the spontaneous ignition of the beech wood accelerated significantly at 300 °C. This indicates the importance of thermal stability studies of polyurethanes used for wooden structure joints. The same research group also showed that the dense beech material (depending on heat treatment conditions) will be heated slowly and the auto-ignition effect will have a long-term character [29]. They indicated that the organics decomposition will be the strongest at 360 °C too. So, it is the end set temperature of the first step of mass loss presented in our paper (Table 2). The authors of the work showed that, at higher temperatures, the decomposition of organics absorbs energy emitted during ignition of the material surface. Thus, overheating of surfaces, corners, edges, and areas close to the polyurethane–wood junction can lead to an acceleration of self-ignition. Therefore, these effects can be intensified and enhanced if we do not control the thermal stability of the PU. Our research on PU adhesive decomposition confirms that the first derivative of the first material mass loss is around 300 °C (Figure 4) and that overlaps with the acceleration of wood auto-ignition at 300 °C. This indicates the importance of our team’s thermal research on the new polyurethane adhesives. Rigid polymers are richer in nitrogen and hydrogen elements in the chain [30]. They proved that rigid resins decompose at temperatures from 200 to 410 °C and 349 flexible ones in the 150–510 °C temperature range. This covers the decomposition temperature range of our various F&R polyurethane adhesives. In our investigations, we used a standard heating rate of 5 °C∙min^−1^. The higher heating rate will shift the onset and maximal decomposition velocity of polyurethanes by a minimum of 50 °C [30] in case of any emergency events. That shift of the thermal degradation of the adhesives with heating rate is connected with the thermal diffusivity and conductivity properties of the materials. In our opinion, the lower the thermal diffusivity and the higher the volume heat capacity are, the higher the degradation temperature changes are. The thermal expansion coefficient is also very important in our research; it influences the thermal properties due to dimensions and density changes of the material in the temperature function. Thermal analysis is also very important because of hazardous products containing nitrogen [31]. In the nitrogen atmosphere, the decomposition stage is up to 450 °C [31]. The supplied energy allows the breaking of the aliphatic bond of the PU to form cyanate and polyol products; also, amine, hydrocarbon, and CO_2_ are formed. At higher temperatures, the list of dangerous compounds such as aldehydes, esters, and N element-containing ones increases. In case oxygen reaches the atmosphere, the char layer (working also as a thermal barrier), which normally protects the material from further pyrolysis reactions, is oxidized. Chen [31] also confirmed that FPU has a wider decomposition range than the RPU materials. In our case, we tried to obtain various F&R PU materials to provide middle thermal properties of FPU and RPU. In comparison to the nitrogen atmosphere, based on our results, PU adhesives lose the majority of the mass in the temperature range of 200–600 °C in oxygen dynamic flow. The oxygen in the atmosphere accelerates the degradation of PU, which was confirmed by He [32] the case of the RPU at higher temperatures and even at a low temperature of 120 °C. By spectroscopic measurements, he confirmed that oxygen influences the kinetics of the partial reaction and the whole degradation process. Per his paper, the degradation range from 200–350 °C is related to polyols, methylene diphenyl diisocyanate (MDI), and its isomers formation. This range corresponds to our TG data called TG 1. At 300 °C, the formation of benzoquinones and nitrogen dioxide begins [32]. Small mass changes below 200 °C and thermal heat flow effects can be associated with water, CH_3_OCH_3_, and unreacted isocyanates. In a non-oxidative atmosphere, oligomers, HCN, MDA, and its isomers, CO_2_, and olefins will be recorded.

### 3.2. Heat Transport Properties of Polyurethane Adhesives

#### 3.2.1. Thermal Expansion of Polyurethane Adhesives

**Figure 5:**
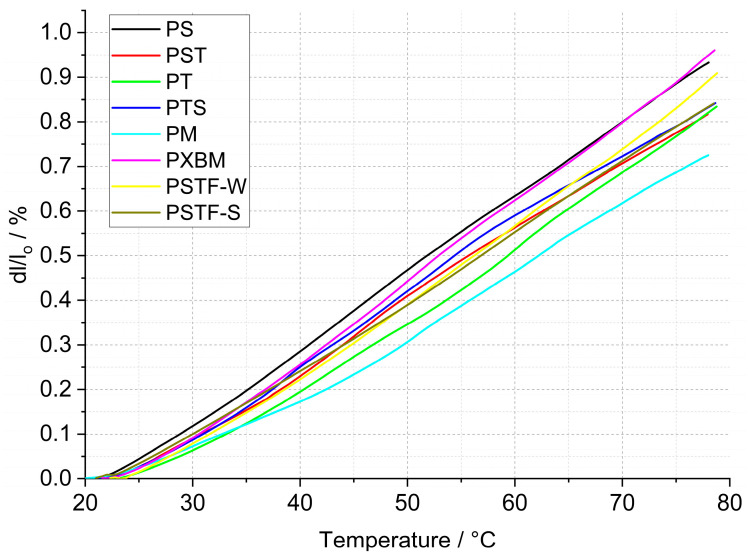
Linear thermal expansion of polyurethanes.

**Figure 6:**
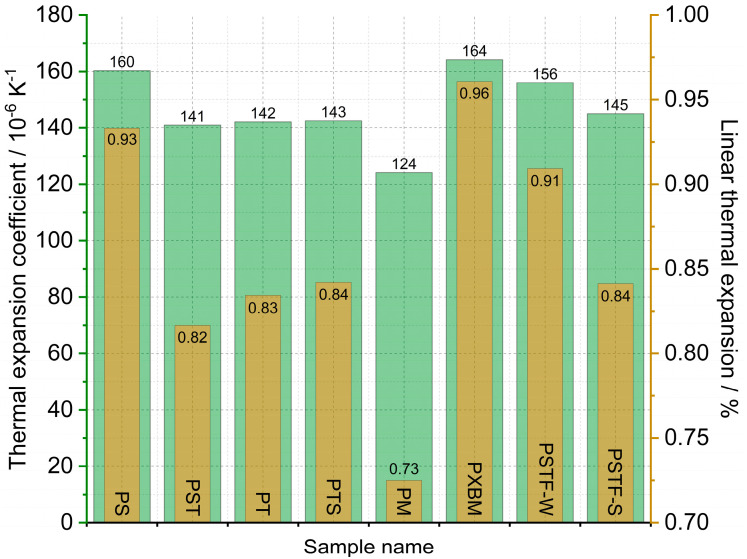
Mean linear thermal expansion coefficient (TEC) and thermal expansion (TE) of the analyzed polyurethanes.

The statistically selected samples of the polyurethanes have been subjected to dilatometric measurement of the linear dimension changes of the adhesives. The calculated linear thermal expansion (TE), based on the recorded data, is shown in Figure 5, and the mean linear thermal expansion coefficient with TE for the 20–80 °C temperature range is presented in Figure 6. The obtained data is planned to be used in future research for the computer simulations of wooden structure joints. The presented data is also used for thermal diffusivity measurement at elevated temperatures to correct the material dimension values.

The data presented in Figure 6 shows that the linear thermal expansion coefficient of the manufactured polyurethane samples is in the range of 124–164∙10^−6^ K^−1^. The lowest TEC value was recorded for the PM material and the highest one for the PXBM one. The linear TE at 80 °C is in the 0.75–0.96% range (Figure 6). The beech wood thermal expansion coefficient versus material direction is similar to the other hardwoods and can reach even 70∙10^−6^ K^−1^ for wet and half of this value for dry material [33], so the best adhesive is the PM resin showing CTE of 124∙10^−6^ K^−1^. Despite such a difference in thermal expansion, flexible polyurethanes are likely to adapt to the dimensions of the wood, which was noticed in our research. The observation of the linear changes curve in the lower temperatures up to 80 °C leads to conclusions concerning the thermal stability of the PU adhesives because any variation on the dilatometric curve will result in mechanical changes of the resin joints in the wooden structure. Thermal expansion is directly connected with the polymer bonds, other thermal properties, and thermal stability, which was discussed by Custódio [34]. That can also be related to the resin degradation accelerated by oxygen, as discussed in Section 3.2.1 of this manuscript.

#### 3.2.2. Thermal Diffusivity and Thermal Conductivity of Polyurethane Adhesives

**Figure 7:**
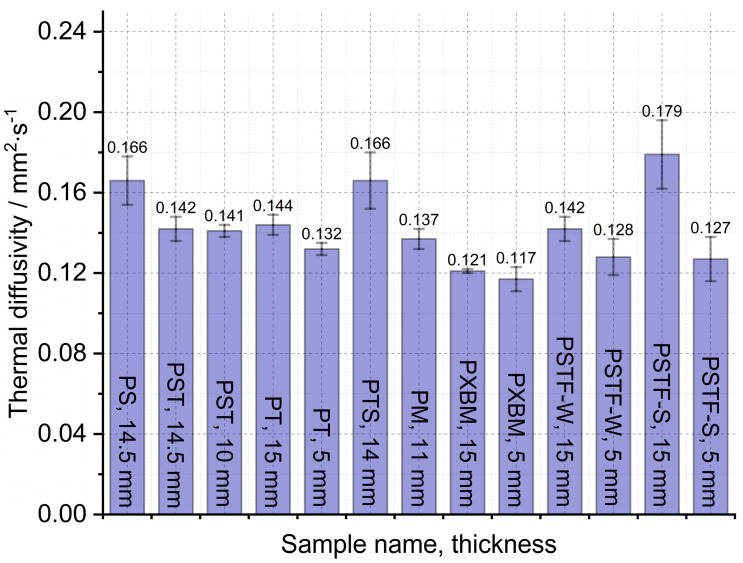
Thermal diffusivity measured on large adhesive samples by Isomet 2114.

**Figure 8:**
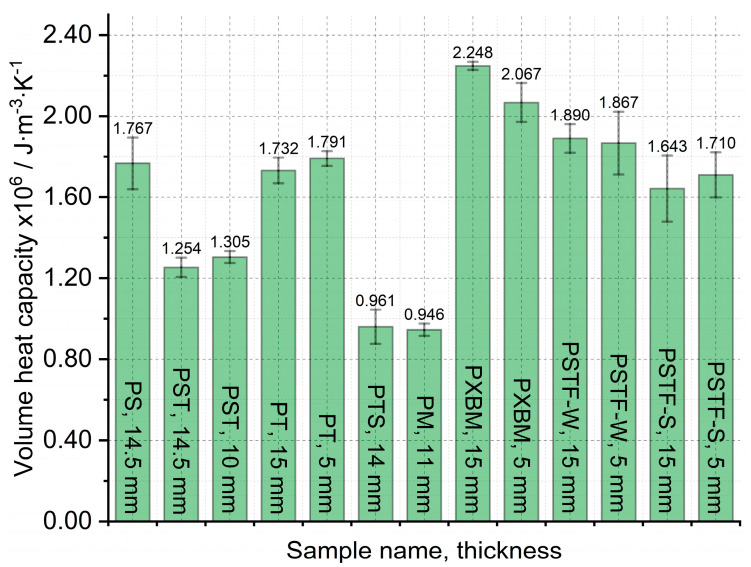
Volume heat capacity measured on large adhesive samples by Isomet 2114.

**Figure 9:**
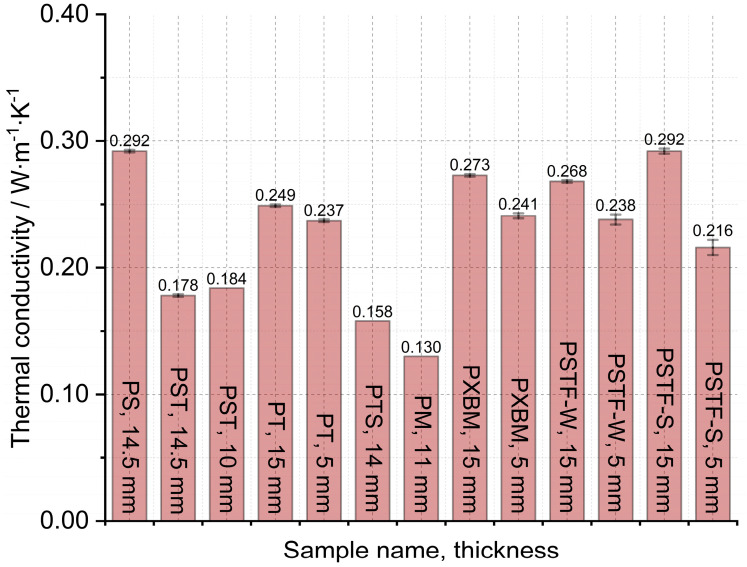
Thermal conductivity measured on large adhesive samples by Isomet 2114.

The large, selected samples were investigated at room temperature using Isomet 2114, which allowed us to analyze the thermal diffusivity, thermal conductivity, and volume heat capacity of the adhesives. The samples were investigated in three different places of materials, and measurements were carried out three times in each place. Such a way allowed for determining the average thermal properties for the whole material and to compare average values in various material measurement positions. The average thermal properties result of the adhesives are shown in Figure 7, Figure 8 and Figure 9.

The obtained results presented in Figure 7 showed that the lowest thermal diffusivity of the adhesives is in the case of the PXBM sample, which is not sufficient due to low thermal stability. Satisfying values were also shown by a new PTSF adhesive, which also presented a good behavior of the mass changes (Figure 3). The rest of the samples had thermal diffusivity of above 0.140 mm^2^∙s^−1^ but not exceeding 0.180 mm^2^∙s^−1^. In the literature, there is only data concerning polyurethanes in foam form but in this paper, we provide an analysis of heat transfer through the material for the application as an adhesive. This is a research novelty in the range of PU adhesives. This parameter is very important for dynamic conditions taking place in case of a fire event and informs us on how the heat will be transferred through the material. So, the lower the thermal diffusivity obtained for the adhesives, the slower the destruction of an adhesive in the larger area of the wood joint. The heat capacity is also very important when it comes to wooden joint applications (Figure 8). The average value of all of the produced adhesives is 1.6∙10^6^ J∙m^−3^∙K^−1^. Close to this value are the PS, PT, and PST resins. These samples also showed good thermal stability close to 200 °C. Except for the PM sample, the thermal conductivity of the adhesives was in the range of 0.16–0.3 W∙m^−1^∙K^−1^ (Figure 9). The obtained heat transfer was lower or similar to the thermal conductivity of the beech wood [35]. This comparison depends on the direction of the measurement in the wood structure but is simultaneously independent of the wood thermal treatment history. The lowest values for tangential direction after the treatment at 220 °C were 0.134 W∙m^−1^∙K^−1^ and in the longitudinal direction equal to 0.478 W∙m^−1^∙K^−1^ for thermally unmodified, respectively [35]. It is known that the lower or similar thermal conductivity of an adhesive to joined wood elements provides the lack of the thermal bridges, which are met in metal joints such as studs (austenitic stainless steel with 16 W∙m^−1^∙K^−1^) and brackets (pre-galvanized mild steel with 44 W∙m^−1^∙K^−1^), which increases the thermal conductivity by at least 18% [36]. This also depends on the direction of the metal joint of the wooden structures. Considering the above, it is believed that the influence of the place and the direction of the wooden structures’ connection will be less sensitive in the case of analyzed PU adhesives.

**Figure 10:**
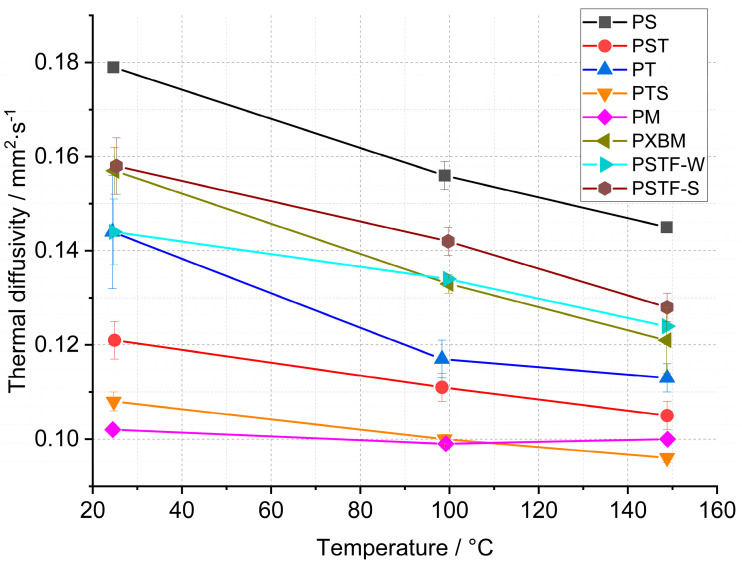
Thermal diffusivity of polyurethane adhesives by LFA method.

**Figure 11:**
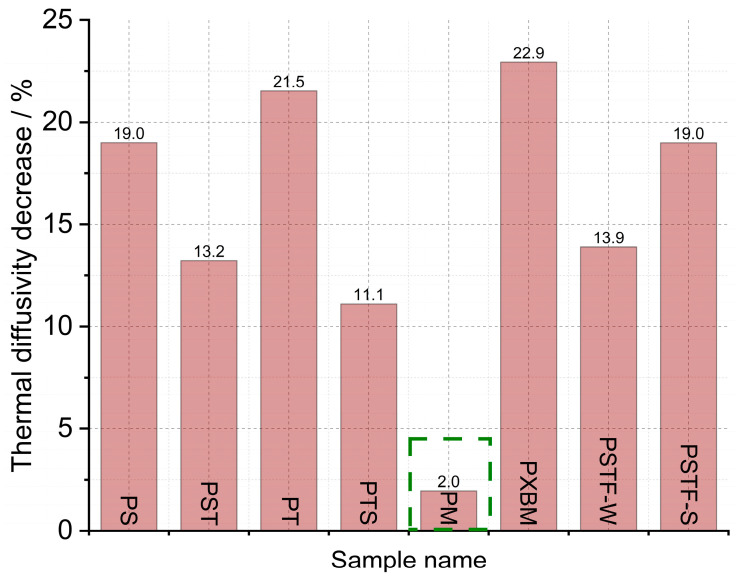
Thermal diffusivity decreases after heating up from RT to 150 °C measured by LFA method.

Due to the requirement of the future computer simulation for wooden constructions, the polyurethane adhesives were subjected to thermal diffusivity measurements by the laser flash analysis method. The results gave information on whether the bulk-prepared adhesive material would be uniform on a smaller scale (10 mm × 10 mm × 3 mm). The obtained results are presented in Figure 10. The presented data gave information concerning the behavior of thermal diffusivity in the function of the temperature, which is presented in Figure 11 as a percentage difference between room and 150 °C temperature.

Material observations after the measurement showed that the PXBM adhesive became soft and unstable at a higher temperature while the other adhesive samples kept their size and stiffness. The results of the thermal diffusivity obtained from the LFA method indicated that the lowest values below 0.130 mm^2^∙s^−1^ are for the PM, PST, and PTS adhesives (Figure 10). In most cases, the LFA method gave different values of this parameter for adhesives in comparison to the recorded data measured on a large scale by Isomet 2114. Only in the case of PS, PT, and PSTF-W, the obtained outcomes were similar. The recognized difference between measurement methods can be decreased by applying pressure while making PU joints between wood parts. By the PM, the decrease in the thermal diffusivity after reaching 150 °C is only 2% (Figure 11). The strongest decrease in heat transfer was around 20% for the PS, PT, PXBM, and PSTF-S materials, which showed the highest thermal diffusivity values at room temperature. Such a high decrease is important to obtain a low thermal property at elevated temperatures. The decreased degree shown in Figure 10 and the slope of the thermal diffusivity changes relate to glass transition and the material structure. The slope of changes will be sharper for much stiffer materials with higher glass transition temperatures. The glass transition temperature for rigid materials usually ranges between −10 and 150 °C. Still, lower temperatures up to −70 °C can show secondary glass transition due to the rotation of the backbone chain named beta relaxation, which is mentioned in the introduction of Raftopoulos’s paper [37]. They confirmed that the glass transition is in elevated temperatures. The rigid PU glass transition above room temperature was also confirmed by other researchers [38]. Flexible PU is in low temperatures, around −40 °C [39]. It is supposed that thermal diffusivity measurement changes versus temperature can be strongly affected by the glass transition of our PU as a mixture of flexible and rigid materials.

## 4. Conclusions

The presented research results of the Flexible PolyUrethanes (FPU) and Rigid PolyUrethanes (RPU) groups allowed the following conclusions to be selected:

The lowest mass decrease was recorded for the PS material with good thermal stability of 200 °C.

- The new PSTF-S/W samples appeared to have good thermal stability temperatures of even 230 °C. These adhesives also have low mass change rates, which is important in case of a fire event.
- The mass change rate DTG analysis showed that this parameter is very important when it comes to the selection of an adequate adhesive that would work in a place where a fire event may appear because of the ventilation of the surroundings.
- The lowest thermal expansion was found for the PM adhesive with the lowest thermal diffusivity in the whole working temperature range.
- For most of the manufactured polyurethane adhesives, the mean thermal expansion coefficient was between 140 and 160∙10^−6^ K^−1^.
- The lowest decrease in thermal diffusivity was recorded for the PM material.
- For most of the investigated polyurethanes, thermal diffusivity was around 0.14 mm^2^∙s^−1^ at room temperature. The highest one was recorded in the case of the PSTF-S one.
- The measurement with the LFA method indicated that the thermal diffusivity drops quickly by 20% to a value below 0.15 mm^2^∙s^−1^ at 150 °C for PU adhesives with high thermal diffusivity at room temperature.
- Considering the good thermal stability, satisfactory thermal conductivity, and thermal expansion, these new polyurethanes, which have not been tested so far regarding the thermal characteristics, will be an excellent alternative to the commonly used adhesives for joining wooden structures.
- It has been proven that the Isomet 2114 apparatus can be used to determine thermal diffusivity and thermal conductivity for large samples of rigid and flexible PU above 20 mm thickness and 70 mm diameter. As demonstrated, such a method will be more precise when samples are not very uniform in small size, and the test is not performed at temperatures higher than 70 °C.
- For higher temperatures, the laser flash analysis method is better. It can give results for high-temperature applications. However, there are two disadvantages: the difficulty of small-size material preparation and statistical selection and the need to measure lots of samples to obtain statistically good results in the case of nonuniform materials on a small scale. The good thing about this method is that it can possibly measure contact resistance between PU and wood, but direction and wood layer should be well selected for future results.
- The LFA measurement will be helpful in our future tests concerning PU thermal analysis of 2–3 mm thick samples taken directly from wooden joints. Such thin samples cannot be measured by Isomet 2114. Still, the LFA can give results for thin samples obtained under various connection pressures and can provide results of thermal contact resistance for specific direction wood joints. PU joints obtained under pressure will be more homogeneous for our future test with the LFA method.
- The DSC-TG data can be used to determine the degradation temperature of PU, which is very important for fire test applications and for setting the measurement range for various analyses.

## Figures and Tables

**Table 1:** Description of various F&R (Flexible and Rigid) polyurethane adhesives [18].

Material Code	Density g∙cm^−3^	Description
PS	1.4	Solvent-free, two-component, polyurethane-based adhesive. Flexible adhesive designed for making flexible joints and for coatings. A:B (100:11)
PST	1.0	Two-component adhesive material based on polyurethanes. Flexible adhesive designed for making flexible for making protective coatings. A:B (100:15)
PT	1.1	Solvent-free, two-component adhesive material based on polyurethanes after hardening. Hard-elastic adhesive, designed for making flexible joints for making protective coatings. A:B (100:52)
PTS	0.9	Two-component adhesive material based on polyurethanes. Permanently elastic adhesive intended for making flexible joints and for making protective coatings. A:B (100:15)
PM	0.9	Two-component adhesive material based on polyurethanes. High-resilience adhesive intended for making flexible joints and for making protective coatings. A:B (100:15)
PXBM	1.4	One-component polyurethane with accelerator, fast-setting while exposed to moisture polyurethane adhesive. Flexible adhesive designed to make flexible joints for the production of protective coatings.
PSTF-W	1.2	Two-component polyurethane construction adhesive that cures at room temperature. It is intended for making flexible joints. A:B (100:100)
PSTF-S	1.21	A two-component polyurethane construction adhesive that cures at room temperature. It is intended for making flexible joints. A:B (100:100)

**Table 2:** Thermal stability and mass changes of polyurethane adhesives.

Material	PS	PST	PT	PTS	PM	PXBM	PSTF-W	PSTF-S
Thermal stability (°C)	200	190	210	200	190	160	200	230
TG 1 (%)	−40.43 200–365 °C	−66.41 190–340 °C	−22.97 210–320 °C	−70.05 200–400 °C	−24.69 190–337 °C	−50.92 160–330 °C	−20.06 200–330 °C	−35.25 230–350 °C
TG 2 (%)	−12.06 365–600 °C	−13.36 340–600 °C	−13.29 320–350 °C	−10.59 400–600 °C	−16.71 337–360 °C	−7.94 330–450 °C	−40.17 330–430 °C	−15.97 350–415 °C
TG 3 (%)	-	-	−22.28 350–430 °C	-	−17.40 360–420 °C	−4.24 450–600 °C	−22.09 430–600 °C	−27.18 415–600 °C
TG 4 (%)	-	-	−28.59 430–600 °C	-	−9.31 420–600 °C	-	-	-
Total TG (%)	−54.24	−78.22	−88.15	−81.72	−69.54	−63.72	−83.77	−79.64

## Data Availability

Data supporting the reported results, including generated data during the study, can be found: https://doi.org/10.58032/AGH/4R9ZZN (accessed on 11 June 2024).
